# Integrated stress response (ISR) activation and apoptosis through HRI kinase by PG3 and other p53 pathway-restoring cancer therapeutics

**DOI:** 10.18632/oncotarget.28637

**Published:** 2024-09-17

**Authors:** Xiaobing Tian, Wafik S. El-Deiry

**Affiliations:** ^1^Laboratory of Translational Oncology and Experimental Cancer Therapeutics, Warren Alpert Medical School, Brown University, RI 02912, USA; ^2^Department of Pathology and Laboratory Medicine, Warren Alpert Medical School, Brown University, RI 02903, USA; ^3^Joint Program in Cancer Biology, Lifespan Health System and Brown University, RI 02903, USA; ^4^Legorreta Cancer Center at Brown University, RI 02912, USA; ^5^Department of Medicine, Hematology/Oncology Division, Lifespan Health System and Brown University, RI 02906, USA

**Keywords:** mutant p53, integrated stress response (ISR), ATF4, HRI, ClpP

## Abstract

Restoration of the p53 pathway has been a long-term goal in the field of cancer research to treat tumors with mutated p53 and aggressive clinical behavior. p53 pathway restoration in p53-deficient cancers can be achieved by small molecules via p53-dependent or p53-independent processes. Hereafter p53-independent restoration of p53-pathway-signaling in p53-deficient/mutated tumors is referred to as ‘restoration of the p53 pathway’. We compare activation of p53 target genes by novel compounds PG3 and PG3-Oc, that activate p53-target genes in a p53-independent manner, and four mutant p53-activating compounds while Nutlin-3a is used as negative control. PG3 and PG3-Oc upregulate p21, PUMA, and DR5 in five cancer cell lines with various p53 mutational statuses through ATF4 (Activating Transcriptional Factor 4) and integrated stress response (ISR) independent of p53. Mutant p53-targeting compounds induce expression of the 3 major downstream p53 target genes and ATF4 in a highly variable and cell-type-dependent manner. PG3 treatment activates ATF4 through ISR via kinase HRI (Heme-Regulated Inhibitor). ATF4 mediates upregulation of PUMA, p21, and NAG-1/GDF15 (Nonsteroidal anti-inflammatory drug-activated gene 1). We note that PUMA mediates apoptosis through activation of caspase-8 in HT29 cells and potentially caspase-10 in SW480 cells. We provide a novel mechanism engaged by PG3 to induce cell death via the HRI/ATF4/PUMA axis. Our results provide unique insights into the mechanism of action of PG3 as a novel cancer therapeutic targeting p53 pathway-like tumor suppression.

## INTRODUCTION

p53-dependent restoration of the p53 pathway can be achieved by small molecules that bind mutant p53, resulting in restoring its wild-type conformation and binding to p53 DNA-binding sites and regulating p53-target gene expression [1–3]. Missense p53 mutants can be divided into two major groups: 1) DNA-contact mutations affecting residues involved directly in DNA contacts without altering p53 conformation, such as hot-spot R273H mutations, and 2) structural mutations that cause a conformational change in the core domain, such as R175H [1].

Toxicity, off-target effects, and limited activity have been roadblocks for small molecules targeting mutant p53 in cancer therapy to progress to the clinic although progress is being made [4]. However, there is still a major unmet medical requirement to target tumors harboring mutant p53. Since thousands of mutations of p53 have been reported [1, 5], the drugs targeting specific mutations may have somewhat limited use in the clinic. In this regard, functional restoration of p53-pathway-signaling using small molecules through other transcription factors, regardless of what kind of p53 mutations exist in the tumor cells, is an attractive method to target mutant p53-bearing tumors. This approach does not aim to restore expression of all p53 target genes through an alternate transcription factor, but instead, it focuses on identifying one or more transcription factors that must positively regulate a key subset of p53 target genes that promote cell cycle arrest and apoptosis. Previously, we reported that ATF4 shares a subset of p53 target genes that are involved in cell cycle arrest, autophagy, and apoptosis [6]. Compound PG3-Oc potently induces cancer cell death through ATF4-mediated restoration of the p53 pathway in various mutant p53-expressing and p53-null cancer cell lines [6]. Our paper provides proof of concept of feasibility and versatility that a small molecule such as PG3-Oc restores p53 pathway signaling in cancer cells in a p53-independent way through ATF4 [6].

Some promising candidate therapeutics have been under development for tumors with mutated p53. The hot-spot mutation R175H causes steric hindrance that reduces affinity for zinc about 100- to 1000-fold, leading to p53 R175H assuming a misfolded conformation [7–10]. ZMC1 (Zinc metallochaperone 1) is a zinc chelator that does not directly bind to mutant p53 but restores a specific class of zinc-binding p53 mutations. Upon treatment with ZMC1, intracellular zinc concentration increases approximately 1000-fold (1.5 nM), and this allows zinc to bind in the p53 R175H native zinc-binding site and restore a wild-type conformation of the mutant p53 (on switch) [7–10]. APR-246 (Eprenetapopt) is a mutant p53-targeting drug that entered a phase 3 clinical trial in 2020 (NCT03745716). Once APR-246 gets into cells, it is converted to the reactive electrophile methylene quinuclidinone (MQ), which binds covalently to the p53 core domain. Cys277 is a prime binding site for MQ in p53 and is essential for MQ-mediated thermostabilization of wild-type, R175H and R273H mutant p53. Importantly, both Cys124 and Cys277 are required for APR-246-mediated R175H mutant p53 reactivation [11, 12]. It has been reported that compound CP-31398 can restore a wild-type DNA-binding conformation of both DNA contact and conformation-changed mutations [13, 14]. Our lab also reported that CP-31398 can stabilize wild-type p53 [15]. However, at this point it is still unclear whether CP-31398 directly binds mutant p53 or not [16]. Ellipticine is an alkaloid isolated from trees of the species *Ochrosia elliptica* and *Rauvolfia sandwicensis*, which inhibits topoisomerase II and leads to DNA damage [17]. Ellipticine induces a shift of mutant p53 conformation towards wild-type. This activity is not due to its ability to inhibit topoisomerase 2. Ellipticine activates the function of both DNA contact and conformation-changed mutant p53, but the mechanisms of this restoration are not known [17]. Nutlin-3a is an E3 ligase MDM2 inhibitor which binds to MDM2 and disrupts MDM2-p53 interaction, thus stabilizing wild-type p53 and activating the p53 pathway [18, 19]. Hence, Nutlin-3a works only in wild-type p53-expressing cells. Of note, ZMC1, CP31398, ellipticine, APR246, and Nutlin-3a also induce cell death through p53-independent mechanisms [10, 14, 20, 21].

The integrated stress response (ISR) is an evolutionarily conserved intra-cellular signaling network and is activated in response to various intrinsic and extrinsic factors. Extrinsic factors include amino acid depletion, glucose deprivation, viral infection, hypoxia, heme deficiency, ROS (reactive oxygen species), DNA damage, and mitochondrial stress [22–26]. Cellular intrinsic stresses, such as ER (endoplasmic reticulum) stress, can also activate the ISR [22]. In the context of cancer biology, oncogene activation, such as MYC overexpression, can trigger the ISR [27]. Cancer cells with enhanced proliferation have enhanced protein synthesis which leads to a high basal level of the ISR as compared to normal cells [27, 28]. This may explain why ISR inducers can selectively target cancer cells. Various stresses are sensed by four specialized kinases (PERK, GCN2, PKR, and HRI) that converge on phosphorylation of serine 51 of eIF2α. eIF2α phosphorylation causes a global reduction of protein synthesis and triggers the translation of specific mRNAs, including ATF4 to help with cell survival and recovery from the stresses [27, 29–31]. However, if the stresses are persistent, ATF4 regulates an apoptosis program to eliminate the damaged cells [22, 32].

ATF4 coordinates a regulation between survival and apoptosis of a cell based on time and exposure to stress. ATF4 has a broad range of control of modulation of immune cells during innate and adaptive responses. Immune cell exposure to a stressed environment in the event of infection, inflammation, and in the tumor microenvironment, results in ATF4 upregulation and activation. ATF4 can regulate the differentiation and maturation of macrophages, T-cells, B-cells, and dendritic cells in tumor immunity [33–35]. Zhang et al. reported that ATF4 is activated following LPS (lipopolysaccharide) stimulation via the TLR4-MyD88 pathway. ATF4 positively regulates expression and secretion of some key inflammatory cytokines, such as IL-6 and IL-8 [36]. Yang et al. reported that ATF4 is important for CD4^+^ T cell activation and proliferation through metabolic reprogramming [37]. Combination treatment of obatoclax with anti-PD-1 antibody synergistically suppresses hepatocellular carcinoma development and prolongs survival of tumor-bearing mice. The combination treatment promotes T-cell activation and effector cytokine expression both in the spleen and tumor [38]. Kim et al. reported that obatoclax decreases human T-regulatory cells (Tregs) while preserving effector T-cells [39]. Obatoclax increases apoptosis of Tregs, profoundly downregulates FOXP3 and CTLA-4 expression, and decreases their suppressive function. Obatoclax increases apoptotic resistance of mature cytotoxic CD8^+^ T-cells [39]. Suresh et al. found that inhibition of heme synthesis leads to HRI activation that enhances PD-L1 translation in NSCLC cells, which can be utilized to sensitize NSCLC to PD-L1 antibody treatment [40].

Because we encountered p53-pathway restoring compounds that did not activate p73 and p63 or that did not lead to mutant p53 downregulation in mutant p53-expressing tumor cell lines, we extended our search for other transcription factors beyond p53 family members. We demonstrated that p53-independent restoration of p53-pathway-signaling can be achieved by small molecules via activation of other transcription factors besides p53 family members p73 or p63 [6]. PG3-Oc is a very potent compound that induces cell death in a wide range of mutant p53-expressing cancer cell lines *in vitro*, and it partially restores the p53 transcriptome through transcription factor ATF4 in a p53-independent manner [6]. Advantages of this approach include that it is not limited by specific p53 single point-mutations, and works in the context of p53-null, p53 deletion, and p53 frameshift mutation carrying cancer cell lines. However, PG3-Oc has poor solubility and is unstable *in vivo*, which limits its antitumor activity *in vivo* [6]. Thus, we have been pursuing pharmacological studies *in vivo* with new PG3-Oc formulations or modifications. Compound PG3 is a derivative of PG3-Oc through removal of its ester group and shortening of its long hydrophobic side-chain while maintaining the pharmacophore of PG3-Oc, which increases solubility in water and stability *in vivo*. With more favorable drug-like characteristics of PG3, we tested whether its structural changes lead to loss of anticancer activity and/or loss of restoration of p53 target genes through ATF4.

Our work provides evidence that PG3 shows the same potency as PG3-Oc, and utilizes the same mechanisms of restoration of p53 target gene activation and ATF4-mediated apoptosis as PG3-Oc. We also report here comparative studies between p53-dependent and p53-independent restoration of the p53 pathway. PG3 treatment triggers the ISR and induces upregulation of ATF4 via HRI, which leads to ATF4-mediated upregulation of p53 target genes important for major functions, p21, PUMA, and NAG-1/GDF15. We provide a novel mechanism by which PG3 leads to p53-independent apoptosis via the HRI-ATF4-PUMA axis. Our results reveal insights into the cancer therapeutic action of PG3.

## RESULTS

### PG3 inhibits the proliferation of cancer cell lines in a p53-independent manner

**Figure 1:**
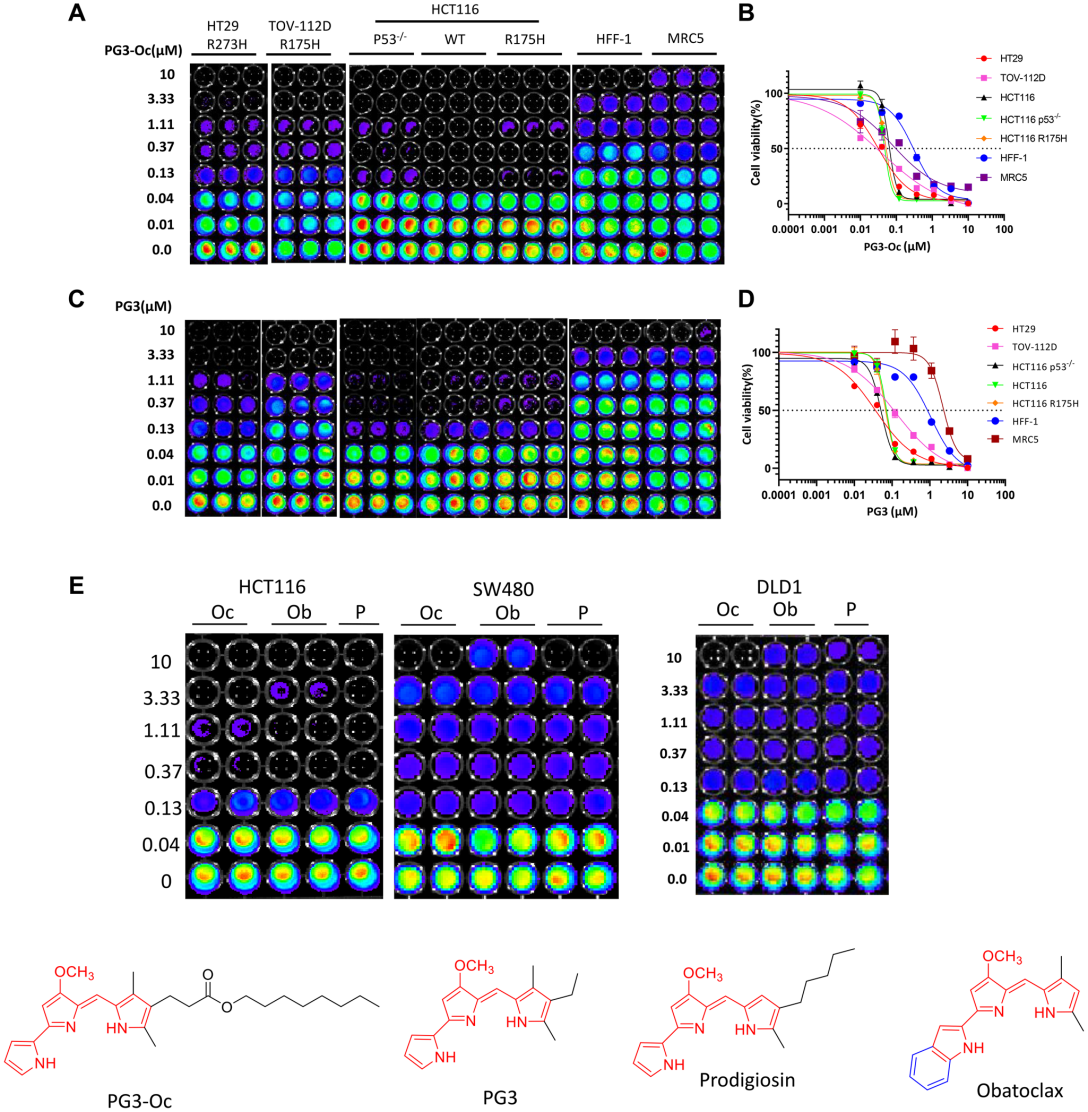
PG3 inhibits cell proliferation and growth in a p53-indepent manner. (**A**–**E**) Cell viability assay, dose response curves and IC_50_ value measurements in a panel of cancer cell lines and normal cells. Cells were treated with different concentrations of PG3, PG3-Oc (Oc), obatoclax (Ob), prodigiosin (P) or DMSO for 72 h. Luciferase activity was imaged by the IVIS Imaging System after treatment. Cell viability data were normalized to those of DMSO treatment control in each cell line and data analyses were performed using PRISM4 software. IC_50_ data are expressed as the mean ±SD (normal; *n* = 3).

To find out if the shortening of the ester group-bearing the hydrophobic side-chain of PG3-Oc will reduce its anticancer activity, cell viability assays were performed using cancer cell lines with p53 mutation R273H (HT29), p53 mutation R175H (TOV-112D), isogenic cell lines HCT116 (wt, p53-null and p53 R175H) and two normal cell lines HFF-1 (human foreskin fibroblast cell with wild-type p53) and MRC5 (human lung fibroblast cell with wild-type p53). The data (Figure 1A–1D and Table 1) indicates that besides TOV-112D, two compounds showed similar potency against the other four cancer cell lines. For TOV-112D, PG3 is 3.3-fold less potent than PG3-Oc. However, PG3’s IC_50_ value is 0.12 and still within the nanomolar range (Table 1). In addition, for normal cell lines HFF-1 and MRC5, PG3 is 3.4-fold and 26.0-fold less toxic than PG3-Oc, respectively (Table 1). For HCT116 isogenic cell lines, both compounds show the same potency, and inhibition of cell viability is independent of p53 status.

**Table 1:** IC_50_ values (μM) of compounds for cancer cell lines with various p53 status and normal cells

	HT29 (R273H)	TOV-1120 (R175H)	HCT116 (P53^−/−^)	HCT116 (WT)	HCT116 (R175H)	HFF-l (WT)	MRC5 (WT)
PG3-Oc	0.031	0.038	0.062	0.047	0.052	0.306	0.078
PG3	0.036	0.126	0.052	0.071	0.066	1.054	2.23
ZMCI	−	−	−	−	−	−	−
CP-31398	0.647	−	−	−	9.578	7.925	5.918
APR-246	−	−	11.300	33.030	8.866	−	−
Ellipticine	0.713	19. 28	2.416	0.999	1.792	6.074	−
Nutalin-3a	−	−	−	−	−	−	−

Symbol “–” indicates that the software cannot calculate a reliable IC_50_ value due to the shapes of the curves.

Taken together, PG3 shows comparable anticancer activity against cancer cell lines that were tested with PG3-Oc causing cancer cell cytoxicity in a p53-independent manner, suggesting the chemical modification does not reduce its anticancer activity. Importantly, PG3 shows significantly less toxicity against normal cells as compared to PG3-Oc, which significantly increases its therapeutic index.

To further support the observation, we include three compounds covering the longest (PG3-Oc), medium (prodigiosin), and shortest (Obatoclax) sidechain-containing compounds with the same pharmacophore (highlighted in red in Figure 1E) for comparison. As shown in Figure 1E, the three compounds show the same potency against colorectal cancer (CRC) cell lines with different p53 statuses, HCT116 (wild-type), SW480 (R273H, P309S), and DLD1 (S241F). In short, the ester group and the long hydrophobic sidechain of PG3-Oc are not required for its anticancer activity.

### p53 pathway-restoring cancer therapeutic compounds inhibit cell proliferation in a p53 status-dependent manner

**Figure 2:**
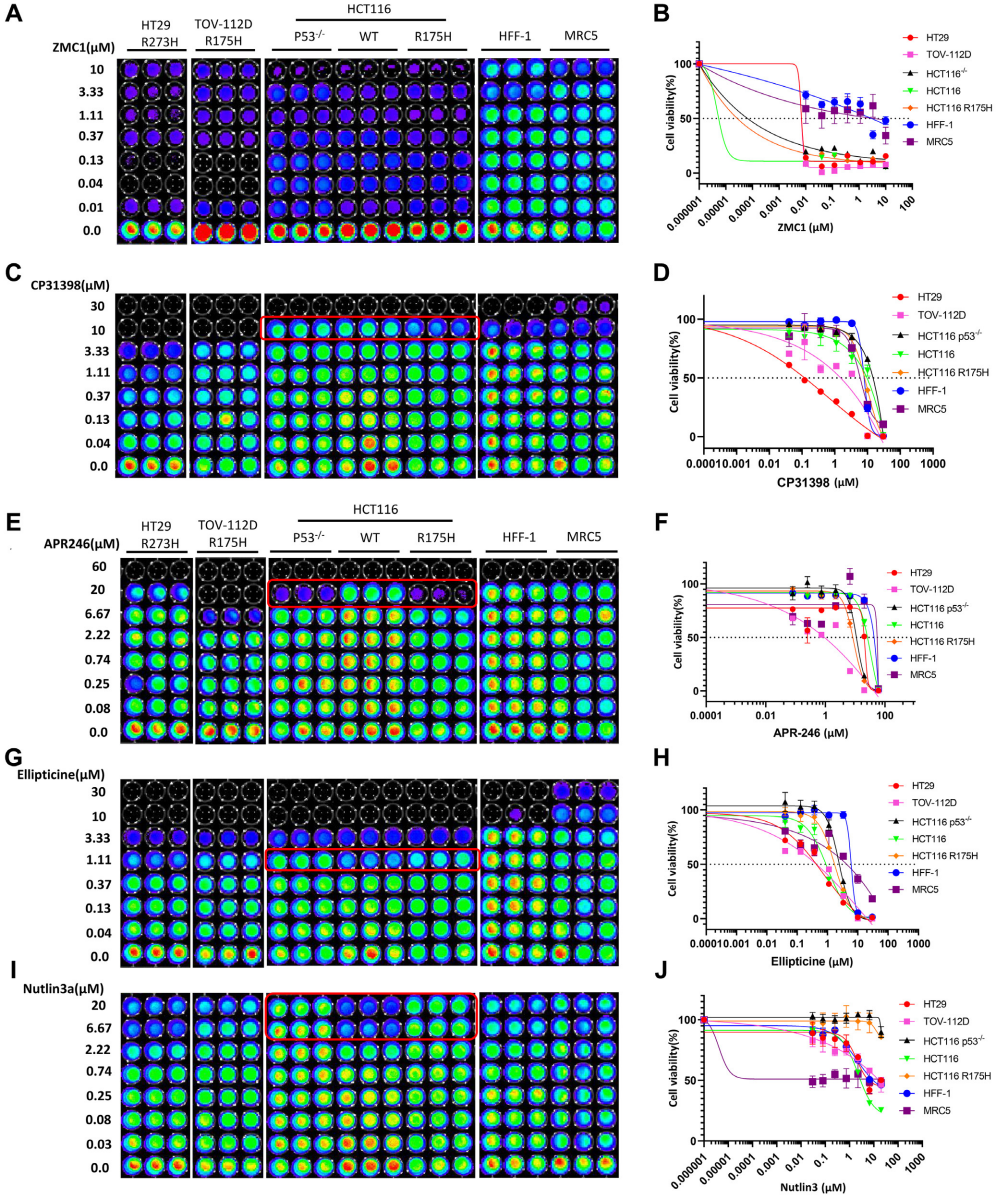
p53-dependent inhibition of cell proliferation is only observed at specific concentrations. (**A**–**J**) Cell viability assay, dose response curves and IC_50_ value measurements in a panel of cancer cell lines and normal cells. Cells were treated with different concentrations of ZMC1, CP-313908, APR-246, Ellipticine, Nutalin-3a or DMSO for 72 h. Luciferase activity was imaged by the IVIS Imaging System after treatment. Cell viability data were normalized to those of DMSO treatment control in each cell line and data analyses were performed using PRISM4 software. IC_50_ data are expressed as the mean ±SD (normal; *n* = 3).

ZMC1 selectively restores R175H conformation-changed p53 mutant at 1 μM concentration and results in p21 upregulation in TOV-112D ovarian cancer cells [10]. However, in our experiment, ZMC1 does not selectively kill TOV-112D cells (Figure 2A, 2B), but instead, it potently inhibits cell viability in all tested cancer cell lines with different p53 statuses even at 0.01 μM. This is possibly because ZMC1 also induces p53-independent cell death [7], but it was much less toxic to HFF-1 and MRC5 normal cells, indicating that the compound has very good selectivity towards cancer cells. The software could not calculate IC_50_ values for each cell line due to the shapes of the IC_50_ curves (Figure 2B and Table 1).

CP-31398 selectively inhibits the growth and proliferation of HT29 (IC_50_ 0.647) and TOV-112D cell lines compared to HFF-1 and MRC5 cells (Figure 2C, 2D, and Table 1). For the HT29 cell line, CP-31398 shows 12.2-fold or 9.1-fold more potency than HFF-1 or MRC5 cells, respectively. HT29 is the most sensitive cell line to CP-31398 among the tested cell lines, and its viability is potently inhibited at a 0.04 μM concentration (Figure 2C). For the three HCT116 isogenic cell lines, selective inhibition of the HCT116-R175H p53-mutated cell line was observed at 10 μM concentration compared to HCT116 (WT) and HCT116 p53^–/–^ cell lines. HCT116 isogenic cell lines are less sensitive to CP-31398 treatment than normal cell lines HFF-1 and MRC5 (Figure 2C, 2D and Table 1) by unknown mechanisms.

The proliferation of both TOV-112D and HT29 cell lines are potently inhibited by APR-246 at 0.08 μM. At this concentration there are no detectable inhibitory effects on HFF-1 and MRC5 (Figure 2E, 2F). TOV-112D is almost completely inhibited and is more sensitive to APR-246 treatment at 6.67 μM as compared to the HT29 cell line (Figure 2E, 2F). At a concentration of 20 μM, p53-null and p53-mutated R175H isogenic HCT116 cell lines are 2.9- and 3.7-fold more sensitive to APR-246 treatment than the p53 wild-type cell line, respectively (Figure 2E and Table 1).

As shown in Figure 2G, 2H, ellipticine treatment from 0.04 to 3.33 μM selectively inhibits the proliferation of HT29 and TOV-112D cells and shows no significant toxicity to HFF-1 and MRC5 cells. For isogenic HCT116 cell lines, ellipticine shows selective toxicity towards the p53-wild-type cell line at 1.11 μM concentration (Figure 2G), suggesting that it is a major event that ellipticine causes DNA damage and activates wild-type p53 as compared to its ability to restore conformation of mutant p53 (R175H).

Nutlin-3a shows inhibitory activity against HT29 and TOV-112D (Figure 2I), which is consistent with Nutlin-3a-induced p53-independent cell death [41]. For the isogenic HCT116 cell lines, Nutlin-3a selectively inhibits the viability of the p53 wild-type cell line and has no significant effects on p53-null and p53 R175H cell lines at a 6.67 μM concentration (Figure 2I, 2J).

### Activation of p53-responsive reporter activity by PG3 and other p53-pathway restoring cancer therapeutic small molecule compounds

**Figure 3:**
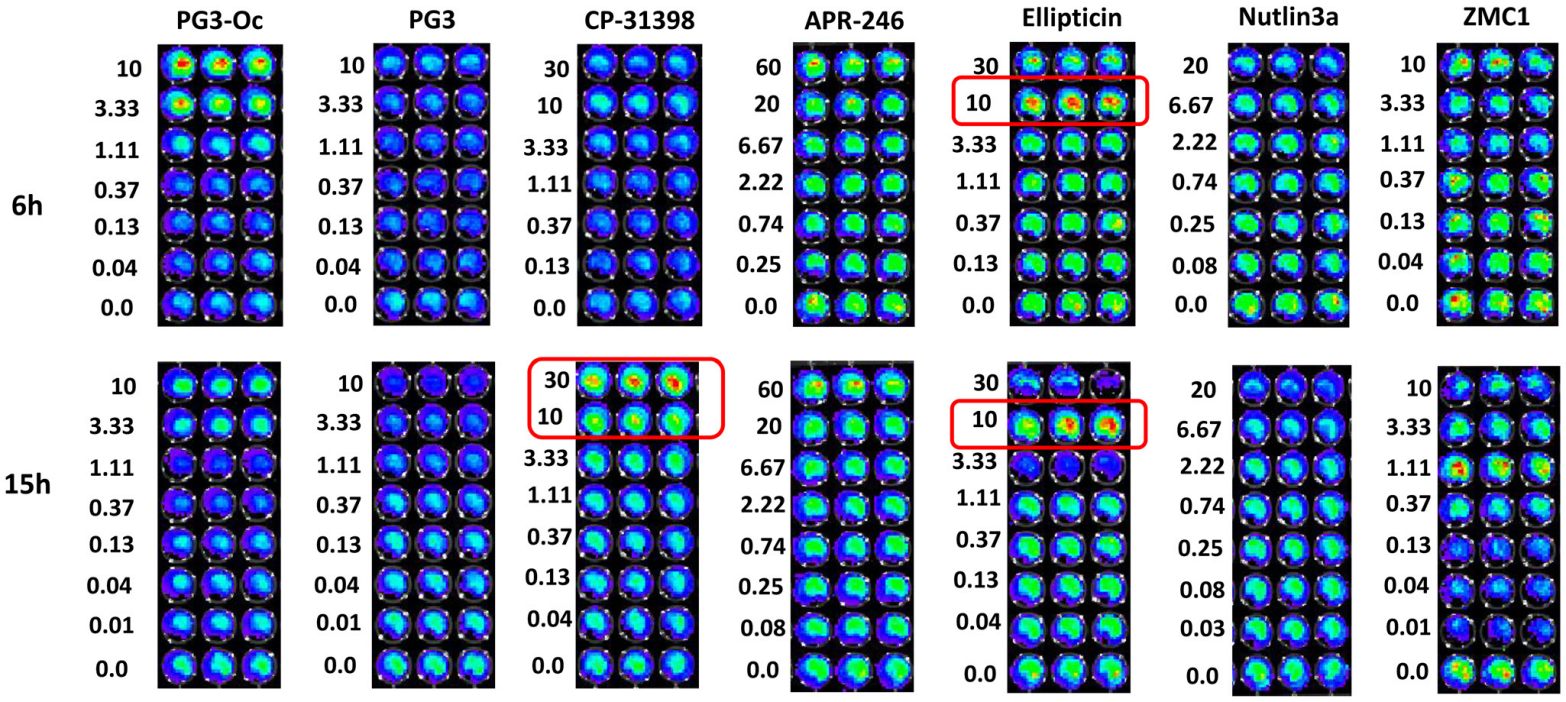
PG3 does not activate p53-responsive reporter activity. SW480 cells carrying a p53-responsive luciferase reporter were used for assay of functional restoration of mutant p53. Cells were seeded in 96-well plates (5 × 10^4^ cells/well) and were treated with compounds indicated for 6 and 15 h, respectively. Then, D-luciferin was added to each well with final concentration 100 μg/mL, and cells were imaged by using an IVIS Imaging System to detect luciferase activity.

To further confirm the ability of p53-dependent or p53-independent restoration of the p53 pathway by the small molecule chemical compounds tested, we performed a p53-responsive reporter assay using p53 mutant cell line SW480 that carries a p53-responsive luciferase reporter (Figure 3). Nutlin-3a was used as a negative control for the assays. At both the 6 hour and 15 hour time points, ellipticine activates the reporter activity at a 10 μM concentration which is in accordance with a previous publication [17]. These results suggest that ellipticine may restore wild-type conformation of p53 with double mutations (R273H/P309S), or it might activate p73 as a paper previously suggested [42].

At 10 μM PG3-Oc treatment for 6 hours there is activation the p53-responsive reporter activity (Figure 3), however, 10 μM is too toxic to all cell lines tested and is also too toxic to normal cells. We never use 10 μM PG3-Oc for studying PG3-Oc-induced apoptosis and p53 pathway restoration. Instead, concentrations used for PG3 and PG3-Oc in our experiments for p53 pathway restoration are 1 μM. Both PG3 and PG3-Oc treatments do not activate reporter activity at 1.11 μM concentration at both the 6 hour and 15 hour time points, suggesting PG3, like PG3-Oc, induces restoration of the p53 pathway is p53-independent [6].

CP-31398 is able to activate the reporter at 30 μM at the 15 hour time point, suggesting the restoration of a wild-type DNA-binding conformation of the mutant p53 (R273H, P309S), or possibly stabilization of p73 [15]. APR-246 is not able to activate the reporter at both the 6 hour and 15 hour time points even when the concentration reaches 60 μM, suggesting it cannot restore wild-type conformation of the mutant p53 (R273H, P309S) that carries double mutations. ZMC1 is not able to activate the p53-responsive reporter at both the 6 hour and 15 hour timepoints, indicating that it is not able to restore wild-type conformation of the mutant p53 (R273H, P309S). This is consistent with previous studies showing it selectively restores conformation-changed mutations of p53, such as R175H.

### PG3 activates ATF4 and upregulates p53 target genes in a p53-independent manner

#### Comparison of the induction of ATF4

**Figure 4:**
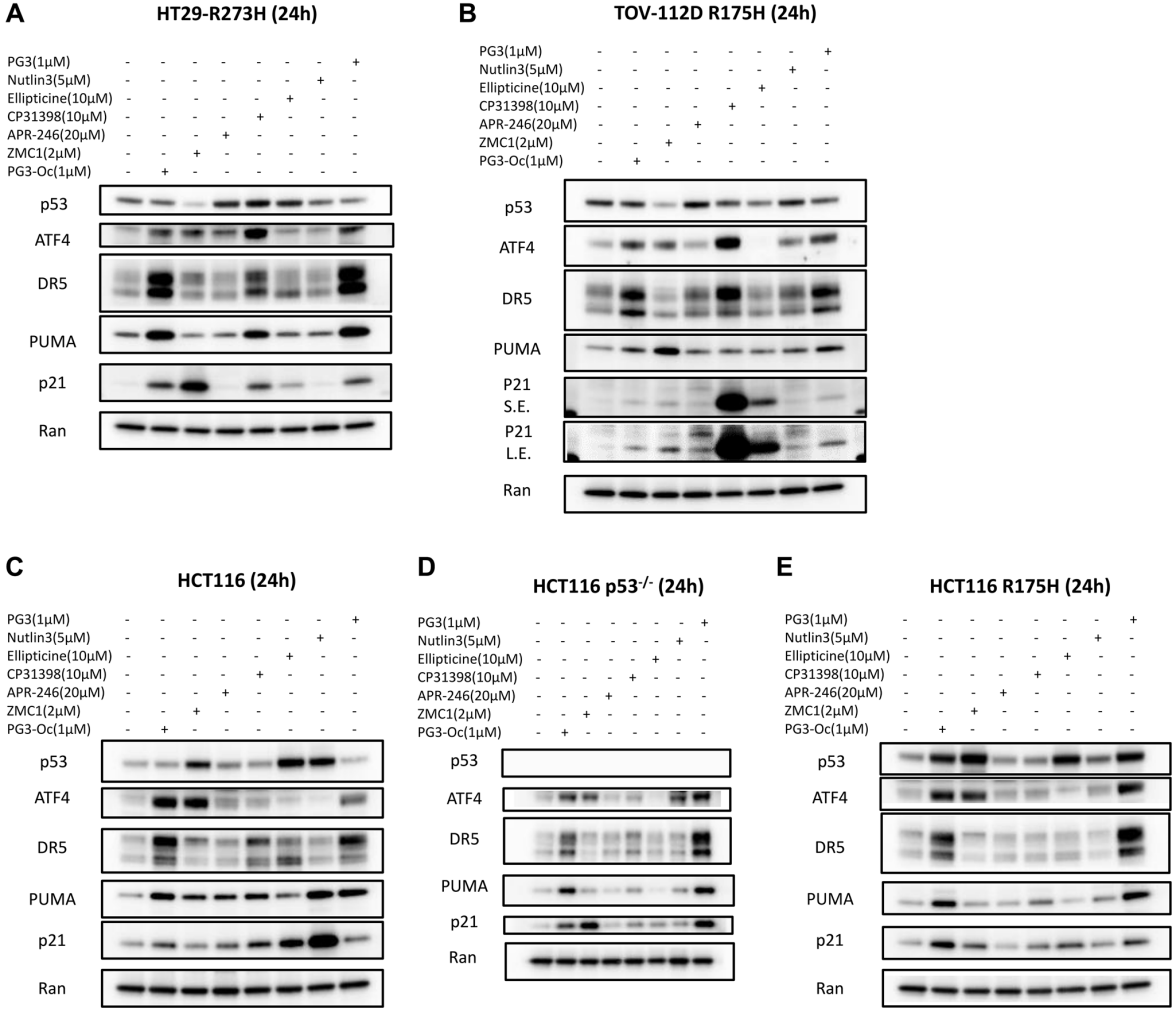
Activation of ATF4 and up-regulation of typical p53 target genes. (**A**–**E**) Western blot analysis of p53 protein levels and upregulation of ATF4, PUMA, DR5 and p21. Cells were treated for 24 h with indicated compounds, concentrations and cell lines. Abbreviations: S.E.: Short exposure; L.E.: Long exposure.

To determine whether PG3 and mutant p53-targeting compounds induce upregulation of ATF4 and typical p53 target genes that regulate cell apoptosis, the five cancer cell lines were treated with the seven small molecule compounds at suitable concentrations for each specific compound for 24 hours, respectively (Figure 4A–4E). Both PG3 and PG3-Oc induce upregulation of ATF4 in the five cell lines significantly and consistently. Interestingly, ZMC1 also induces ATF4 expression to almost the same levels as PG3 and PG3-Oc in each of the five cell lines (Figure 4A–4E). By contrast, APR-246 does not upregulate ATF4 in each of the five cell lines. CP-31398 more potently induces upregulation of ATF4 in HT29 and TOV-112D cell lines as compared to PG3 and PG3-Oc but does not upregulate ATF4 in the three isogenic HCT116 cell lines, indicating that CP-31398 upregulation of ATF4 is cell type-dependent. Ellipticine and Nutlin-3a do not upregulate ATF4 in all of the tested cell lines.

#### Comparison of p53 stabilization by PG3 and other p53 function restoring small molecules

PG3 and PG3-Oc show no effects on stability of endogenous mutant p53 protein (Figure 4A, 4B). They also do not upregulate expression of wild-type p53 protein (Figure 4C). However, PG3 and PG3-Oc stabilize exogenous mutant p53 R175H in engineered HCT116-R175H cells (Figure 4E) that overexpresses the p53-R175H mutant. ZMC1 significantly downregulates both endogenous R175H and R273H mutant p53 proteins, the latter of which has not previously been reported (Figure 4A, 4B). By contrast, ZMC1 stabilizes exogenous R175H mutant p53 (Figure 4E). Interestingly, ZMC1 also stabilizes wild-type p53 (Figure 4C) which has also not been previously reported.

APR-246 stabilizes endogenous mutant p53 R273H and R175H significantly (Figure 4A, 4B), which is consistent with previous publications [11, 12]. But it does not stabilize exogenous R175H mutant p53 (Figure 4E). Moreover, APR-246 shows no effect on the level of wild-type p53 protein (Figure 4C). CP-31398 treatment stabilizes the endogenous R273H p53-mutant, and marginally destabilizes the R175H p53-mutant (Figure 4A, 4B). By contrast, CP-31398 has no effects on the level of exogenous R175H p53-mutant protein (Figure 4E). Also, CP-31398 shows marginal stabilization of wild-type p53 (Figure 4C). Ellipticine treatment stabilized the endogenous R273H p53-mutant, and mildly downregulates the endogenous R175H p53-mutant protein (Figure 4A, 4B). However, ellipticine upregulates the exogenous R175H p53-mutant (Figure 4E) and potently stabilizes wild-type p53 (Figure 4C), which is consistent with its DNA damage property. As expected, Nutlin-3a has no effects on the protein levels of endogenous and exogenous p53 mutants (Figure 4A, 4B, 4E), and stabilizes wild-type p53 (Figure 4C).

#### Comparison of induction of three typical p53 target genes by PG3 and other p53 pathway restoring small molecule compounds

We compared expression of three major p53 target genes that regulate cell apoptosis. Both PG3 and PG3-Oc consistently induce upregulation of DR5, PUMA, and p21 in the five cell lines, which is consistent with the interpretation that PG3, like PG3-Oc, regulates the expression of a subset of p53 target genes (Figure 4A–4E).

ZMC1 treatment does not induce the upregulation of DR5 in the five cell lines though it does upregulate ATF4 in each tested cell line (Figure 4A–4E). ZMC1 does not upregulate PUMA, but potently induces p21 in the HT29 p53 mutated R273H cell line (Figure 4A). By contrast, ZMC1 upregulates PUMA and slightly upregulates p21 in the TOV-112D-R175H cell line (Figure 4B). However, for HCT116 p53-mutaant RI75H cells, ZMC1 mildly induces p21, but does not upregulate PUMA (Figure 4E). ZMC1 upregulates PUMA, but not p21 in the p53-wild-type HCT116 cells. By contrast, ZMC1 upregulates p21, but not PUMA in p53-null-HCT116 cells (Figure 4C, 4D). The data suggest that ZMC1-mediated restoration of the p53 pathway is variable and dependent on cell types and p53 mutations. ZMC1-mediated PUMA induction is dependent on p53 as it induces upregulation of PUMA in HCT116 cells, but not in HCT116 p53^–/–^ cells. We chose 20 μM of APR-246 for studies of induction of p53 target genes because APR-246 selectively inhibits cell proliferation and leads to cell death of p53-null and p53-R175H cell lines at this specific concentration (Figure 2E). Like ZMC1, APR-246 treatment does upregulate DR5 in the five cell lines (Figure 4A–4E). APR-246 upregulates PUMA only in the p53 wild-type HCT116 cell line, but not in HT29, TOV-112D, HCT116 p53-mutant R175H, and HTC116 p53^–/–^ cell lines. It slightly induces p21 in R175H TOV-112D and p53 wild-type HCT116 cell lines, but not other cell lines (Figure 2E). CP-31398 mildly upregulates DR5 in the HT29 cell line, but the induction is less potent than with PG3 and PG3-Oc. By contrast, CP-31398 upregulates DR5 in a comparable manner to PG3 and PG3-Oc in TOV-112D cells. CP-31398 does not upregulate DR5 in the three isogenic HCT116 cell lines (Figure 4A–4E). CP-31398 induces PUMA expression in HT29 and p53-wild type HCT116 cells but barely in TOV-112D, p53-null, and R175H-expressing HCT116 cells. CP-31398 upregulates p21 strongly in TOV-112D cells and significantly in HT29 and p53 wild-type HCT116 cells, but barely in p53-null and p53-R175H HCT116 cells. Taken together, our results suggest that CP-31398-induced restoration of mutant p53 tumor suppressor function and expression of p53 target genes is also cell type-dependent. CP31398 does not restore the function of exogenous mutant p53 R175H (Figure 4E). Ellipticine slightly upregulates the short isomer of DR5 in HT29 and HCT116 cells, but not in the HCT116 p53^–/–^, HCT116 p53-R175H, and TOV-112D cell lines (Figure 4A–4E). Ellipticine does not upregulate PUMA in HT29 and TOV-112D cells while it mildly upregulates PUMA in the HCT116 cell line, but not in the HCT116 p53^–/–^ and HCT116 p53-R175H cell lines. Ellipticine slightly upregulates p21 in the HT29 and HCT116 p53^–/–^ cell lines, mildly upregulates p21 in TOV-112D and HCT116 p53-R175H cell lines, and significantly upregulates p21 in HCT116 cells. The results are consistent with the cell viability assays showing that ellipticine selectively inhibits the proliferation of p53 wild-type HCT116 cells (Figure 2G). Our data suggest that ellipticine stabilization of wild-type p53 through DNA damage might dominate over its restoration of mutant p53 in the isogenic cell lines. Nutlin-3a does not upregulate DR5 in five cell lines and significantly upregulates PUMA and p21 only in the p53 wild-type HCT116 cell line (Figure 4A–4E), which is consistent with its known inhibition of MDM2 and stabilization of wild-type p53.

In summary, PG3 and PG3-Oc induce significant upregulation of p21, PUMA, and DR5 consistently in the five tested cancer cell lines with various p53 statuses in a p53-independent manner. However, the other compounds tested which target mutant p53 and restore its wild-type conformation, induce the expression of the three p53 target genes in a highly variable and highly cell type-dependent way.

### PG3 induces apoptosis in p53-null and p53-mutant cancer cell lines

**Figure 5:**
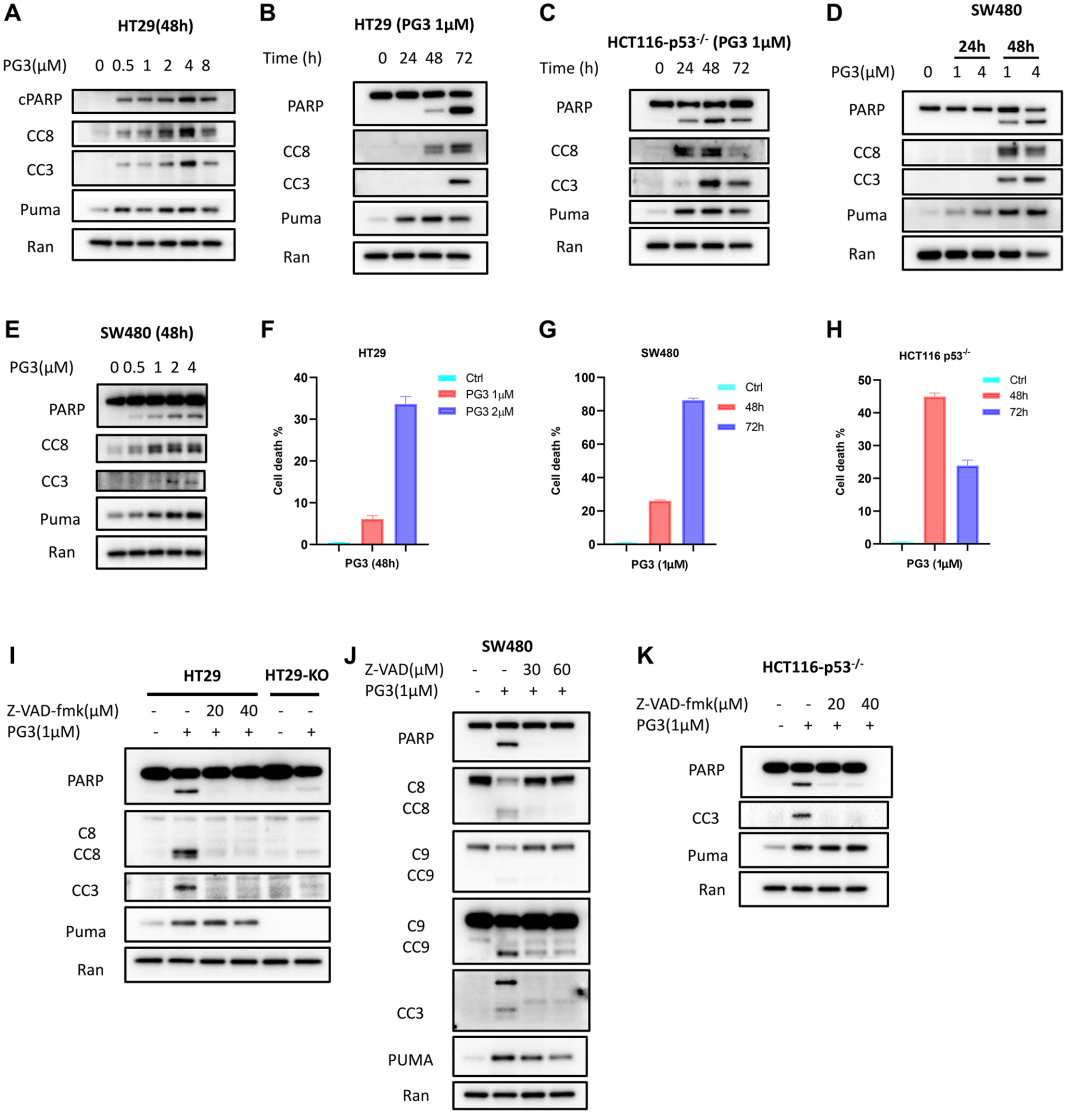
PG3 induces apoptosis in p53-null and p53-mutant cancer cell lines. (**A**–**E**) Dose-response and time-course analysis of cleavage of caspase-8, -3, cleaved PARP and PUMA in PG3-treated HT29, SW480 and HCT116 p53^−/−^ cells by western blot using the indicated antibodies. (**F**–**H**) Flowcytometry analysis of cell death. (**I**) HT29 and HT29-PUMA^−/−^ and (**J**) SW480 cells were co-treated with PG3 and pan-caspase inhibitor Z-VAD-fmk for 48 h. (**K**) HCT116 p53^−/−^ cell were co-treated with PG3 and Z-VAD-fmk for 24 hours.

To evaluate if PG3-induced cell death is caspase-dependent, apoptosis markers were analyzed by western blot. Dose-response experiments show that as low as 0.5 μM PG3 is sufficient to induce PUMA and activate cleaved caspase-8 and -3 and cleaved-PARP in HT29 and SW480 cells (Figure 5A, 5D, 5E). Time-course experiments indicate that PUMA protein is first induced at 24 hours and this induction is sustained even at 48 hours and 72 hours (Figure 5B–5E). At 48 hours, we note that cleavage of PARP, as well as caspase-8 and -3, occur in HT29, HCT116 p53^–/–^ and SW480 colorectal cancer (CRC) cell lines (Figure 5B–5E). These data indicate that PG3-induced upregulation of PUMA is earlier than cleavage of PARP, caspase-8, and -3, which occurs in a dose- and time-dependent manner.

Flow cytometry cell death analysis was performed using Zombie Violet dye. Zombie Viole is an amine-reactive fluorescent dye that is non-permeant to live cells but permeant to the dead cells with compromised membranes. HT29, SW480 and HCT116 p53^–/–^ cells were treated with PG3 for different concentration and time, percentage of dead cells were analyzed as shown in Figure 5F–5H. As to HCT116 p53^–/–^ cells. At 72 h time point, a great amount of dead HCT116 p53^–/–^ cells turned into cell debris as flow cytometry showed the population of cell debris significantly increased compared to 48 h treatment. Because we gated singlets for cell death analysis, therefore, 72 h treatment showed less dead cell population than 48 h.

Caspase-dependent induction of apoptosis is further confirmed by the pan-caspase inhibitor (Z-VAD-FMK) co-treatment experiments with PG3. Western blot analysis shows that Z-VAD-FMK potently inhibits the cleavage of PARP, caspase-8, and -3 in HT29, SW480, and HCT116 p53^–/–^ cells (Figure 5I–5K). Taken together, these data suggest that PG3 treatment induces caspase-8 and caspase-3 activation in CRC cell lines, and caspase activation is required for PG3-induced cell death.

### ATF4 is a key regulator in mediating PUMA upregulation that is required for PG3-induced apoptosis

**Figure 6:**
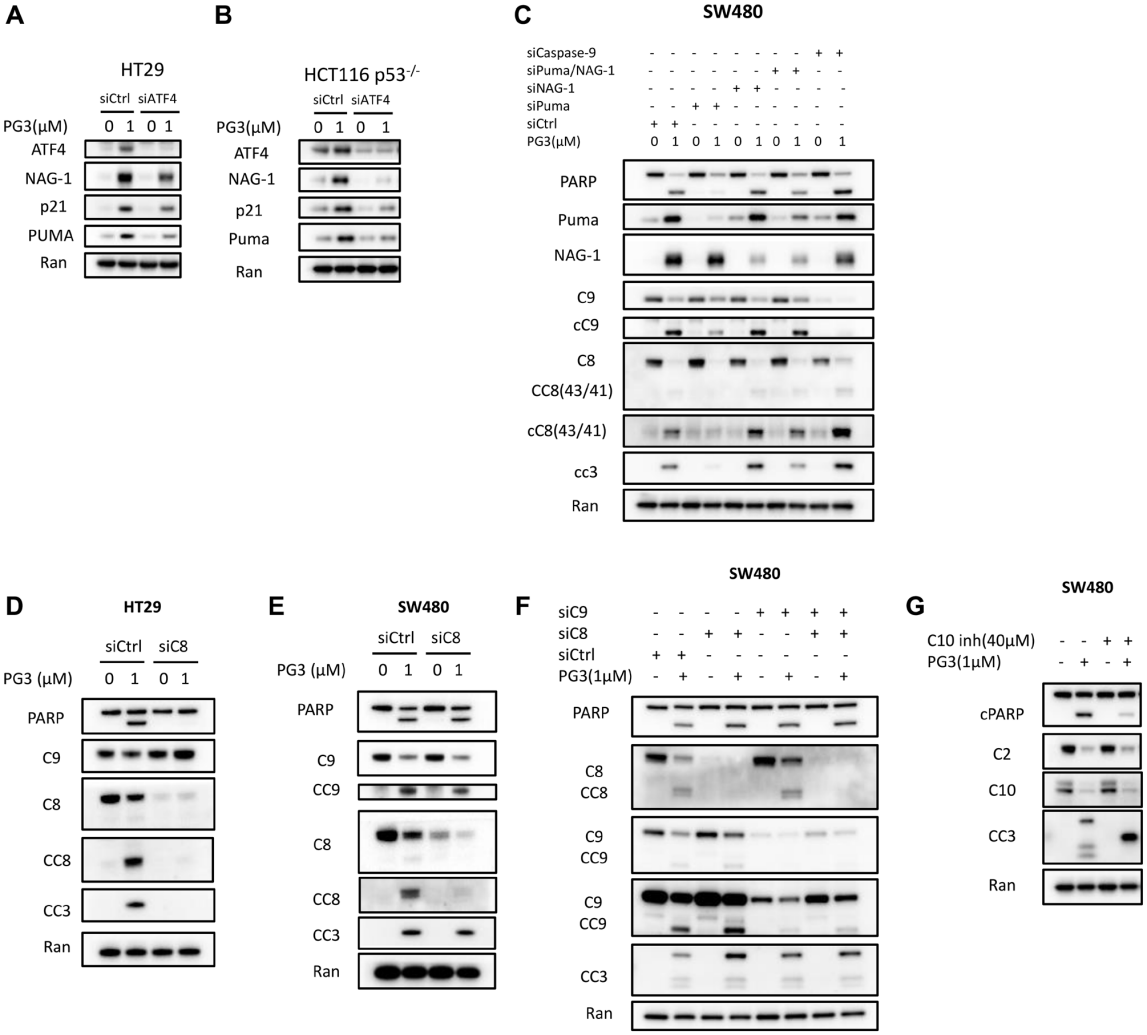
ATF4 is a key regulator and mediates PUMA expression that is required for PG3-induced apoptosis. (**A**) HT29 and (**B**) HCT116 p53^−/−^ cells were transfected with Control and ATF4 siRNAs, and at 32 hours after transfection, the cells were treated with 1 μM PG3 for 16 h. Then cell lysates were prepared, and Western blotting was performed using the indicated antibodies. (**C**) SW480 cells were transfected with siControl, siPUMA, siNAG-1, siCaspase-9 for 24 hours, and then treated with PG3 for 24 hours. Western blots were performed using the indicated antibodies. (**D**) HT29 and (**E**) SW480 cells were transfected with siControl, siCaspase-8 for 24 hours and then treated with PG3 for 48 and 24 hours, respectively. (**F**) SW480 cells were transfected with siControl, siCaspase-8 and siCaspase-9 for 24 hours and then treated with PG3 for 24 hours. Western blots were performed using the indicated antibodies. (**G**) SW480 cells were co-treated with PG3 and caspase-10 inhibitor for 24 hours, and then Western blots were performed using the indicated antibodies.

We previously identified ATF4 as a key transcription factor that regulates PG3-Oc-induced upregulation of a subset of p53-target genes, including PUMA which plays an important role in PG3-Oc-induced apoptosis [6]. NAG-1 (also known as GDF15) is a putative tumor suppressor whose expression can be induced by drug treatment [43]. NAG-1/GDF15 is both a p53 and an ATF4 target gene [6] and a pro-apoptotic protein. A previous report indicates that prodigiosin-induced upregulation of NAG-1/GDF15 mediates caspase-9 activation through mitochondrial and cellular apoptosis [44]. Here, we find that knockdown of ATF4 significantly reduces PG3-induced upregulation of PUMA, p21, and NAG-1/GDF15 in both the HT29 and HCT116 p53^–/–^ cell lines (Figure 6A, 6B).

PUMA is a BH-3-only Bcl-2 family member that binds and inactivates anti-apoptotic proteins of Bcl-2, Bcl-X_L_, and Mcl-1. This facilitates induction of caspase-9 mediated intrinsic apoptosis pathway [45, 46]. Since PUMA is an important proapoptotic protein, we evaluated if PUMA is required for PG3-mediated cell death in *PUMA* gene knockout cell line HT29-PUMA^–/–^ [6]. As seen in Figure 5F, there is strong inhibition of PG3-induced cleavage of caspase-8, caspase-3, and PARP. Similar results are observed in SW480 cells by knockdown of PUMA using siRNA (Figure 6C), which not only potently blunts cleavage of caspase-8, -3, and PARP, but also cleavage of caspase-9 which is consistent with our previous observations [6]. Taken together, these data suggest that PUMA protein is required and is a key mediator for cell death induced by PG3 treatment in the HT29 and SW480 cell lines.

PG3 treatment does not activate caspase-9 in HT29 cells (Figure 6D) but knockdown of caspase-8 blocks cleavage of caspase-3 and PARP (Figure 6D). This is not the case in SW480 cells (Figure 6E). Knockdown of caspase-8 does not prevent cleavage of caspase-3, caspase-9, and PARP. We hypothesize that PG3-induced apoptosis is mediated by caspase-9 in SW480 cells. However, knockdown of caspase-9 (Figure 6C, 6F), double knockdown of caspase-8 and caspase-9 (Figure 6C), and knockdown of NAG-1/GDF15 (Figure 6C), does not prevent cleavage of caspase-3 and PARP. To search for caspases that mediate caspase-3 activation in the SW480 cell line, we selected caspase-10 because it is an initiator caspase upstream of caspase-3 and it was reported that taxol induces caspase-10-dependent apoptosis [47]. As seen in Figure 6G, a caspase-10-specific inhibitor significantly reduces PG3-induced cleavage of caspase-3 and PARP in SW480 cells. This suggests that PUMA might mediate activation of caspase-10 in SW480 cells since knockdown of PUMA prevents PG3-induced cleavage of caspase-3 and PARP.

### PG3 induces upregulation of ATF4 through ISR via HRI

**Figure 7:**
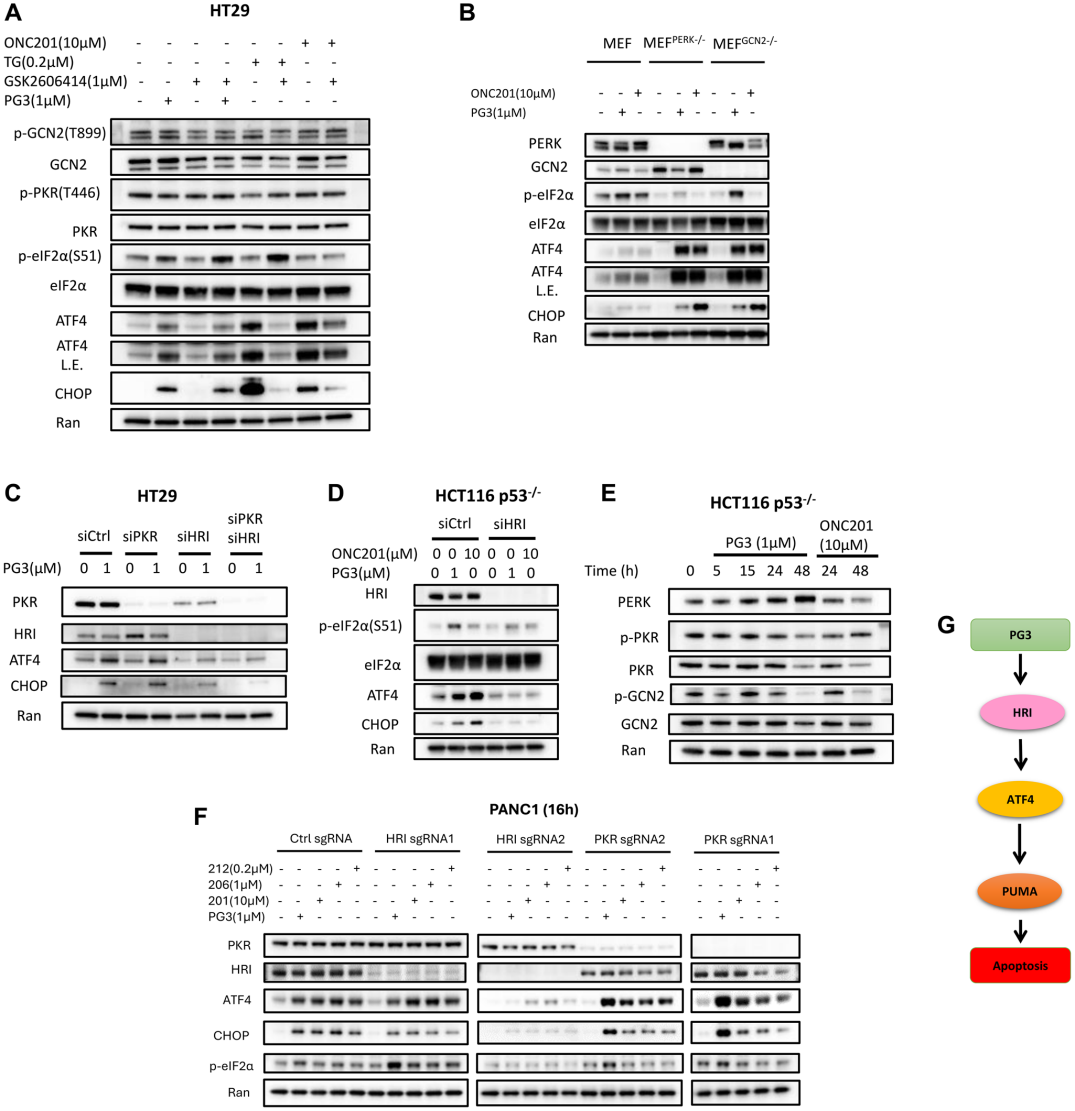
PG3 induces upregulation of ATF4 through ISR via HRI. (**A**) HT29 cells were treated with PG3, GSK2606414, TG and ONC201 respectively, or co-treatment with PG3/GSK2606414, PG3/TG, and PG3/ONC201 for 15 h. (**B**) MEF, MEF^PERK−/−^ and MEF^GCN2−/−^ cells were treated with PG3 and ONC201 for 16 hours. (**C**) HT29 cells were transfected with siControl, siPKR, siHRI and siPKR/siHRI for 24 hours and then treated with PG3 for 16 hours. (**D**) HCT116 p53^−/−^ cells were transfected with siControl and siHRI for 24 hours and then treated with PG3 for 16 hours. (**E**) HCT116 p53^−/−^ cells were treated with PG3 and ONC201 for indicated times. (**F**) PANC1-sgCtrl, -sgHRI and -sgPKR cell lines were treated with PG3 and imipridones (ONC201, ONC206 and ONC212) for 16 hours. Western blot was performed using indicated antibodies. (**G**) Proposed model of PG3-induced cell apoptosis through HRI/ATF4/PUMA axis. Abbreviation: L.E.: Long exposure.

Previously we reported that PG3-Oc-induced upregulation of ATF4 was not through ER stress and PERK (PKR-like endoplasmic reticulum kinase), but upstream factors of ATF4 were not identified [6]. To explore upstream regulators of ATF4, two controls were used. One is thapsigargin (TG) which is a known ER stress inducer that activates PERK, and another is ONC201 which induces ISR through HRI [48, 49]. As seen in Figure 7A, PG3 or ONC201 treatment does not increase phosphorylation of GCN2 (General control non-depressible 2) and PKR (Protein kinase R), suggesting GCN2 and PKR are not activated by PG3 or ONC201. We did not probe phospho-HRI because phospho-HRI antibodies are not commercially available. The PERK inhibitor GSK2606414 does not block increased phosphorylation of eIF2α, upregulation of ATF4 protein, and its target gene CHOP. By contrast, the PERK inhibitor abolishes TG-induced upregulation of ATF4 and CHOP. This suggests that PG3 treatment also does not induce ER stress, therefore, PG3-induced upregulation of ATF4 is not through kinase PERK. To further verify those observations, MEF^PERK–/–^ and MEF^GCN2–/–^ cell lines are treated with PG3, PERK inhibitor, and ONC201. Knockout of *PERK* or *GCN2* genes does not abolish PG3- or ONC201-induced upregulation of ATF4 and CHOP (Figure 7B). Taken together, PG3-induced upregulation of ATF4 is not through PERK and GCN2.

Interestingly, the PERK inhibitor significantly attenuates ONC201-induced upregulation of ATF4 and CHOP (Figure 7A) because it turns out that GSK2606414 also potently inhibits HRI (IC_50_ 0.42 μM) [50]. To study whether PKR and/or HRI mediates PG3-induced upregulation of ATF4, siRNA knockdown experiments were performed. knockdown of PKR does not prevent ATF4 and CHOP from upregulation (Figure 7C), which is consistent with the fact that PG3 treatment does not activate PKR as the phosphorylation level of PKR does not increase as compared to untreated controls (Figure 7A, 7E). Knockdown of HRI blocks ATF4 and CHOP upregulation as compared to the untreated control. Double knockdown of PKR and HRI shows the same effects on ATF4 and CHOP induction as knockdown of HRI alone in the HT29 cell line (Figure 7C). These observations are further confirmed by using another cell line HCT116 p53^–/–^ and ONC201 as a positive control, that is, knockdown of HRI blocks PG3- or ONC201-induced upregulation of ATF4 and CHOP (Figure 7D).

To further verify that PG3 activates ISR through HRI, *HRI* and *PKR*-KO cell lines were treated with PG3 and imipridones for 16 hours respectively (Figure 7F). Western blot indicated that knockout of PKR gene had no effect on PG3 and imipridones-induced upregulation of phosphorylation of eIF2α (S51), ATF4 and CHOP. sgHRI#1 knockout of HRI gene is not perfect as western blot showed small amounts of HRI, therefore it did not block induction of phosphorylation of eIF2α (S51), upregulation of ATF4 and CHOP. sgHRI#2 knockout of HRI gene efficiently, it potently reduced the phosphorylation of eIF2α (S51), and upregulation of ATF4 and CHOP (Figure 7F). These data are consistent with siRNA knockdown results, and further support that PG3 triggers the ISR through kinase HRI.

In summary, we propose a model of PG3-induced apoptosis in Figure 7G. PG3 treatment triggers the ISR with subsequent upregulation of ATF4 via HRI. ATF4 positively regulates PUMA gene expression which then leads to apoptosis.

### Inhibition of heme biosynthesis leads to upregulation of ATF4

OMA1 (Overlapping with the M-AAA protease 1 homolog), is a mitochondrial stress-activated protease, and DELE1 (DAP3-binding cell death enhancer 1) is a little-characterized protein that is associated with the inner mitochondrial membrane. Recently two papers published in Nature reported that mitochondrial stress induced by compound CCCP (carbonyl cyanide m-chlorophenyl hydrazone) stimulates OMA1-dependent cleavage of DELE1 and leads to the release and accumulation of a short form of DELE1 in the cytosol, where it binds and activates HRI independent of heme [25, 26]. Since PG3-Oc treatment causes mitochondria stress [6] and ONC201 induces mitochondria stress by binding and activating mitochondria protease ClpP [51], we were interested in determining whether both PG3 and ONC201 activate HRI through the OMA1/DELE1/HRI pathway. siRNA knockdown of OMA1 or DELE1 were performed as shown in Supplementary Figure 1A–1D. OPA1 (Optic atrophy protein 1 or mitochondrial dynamin-like GTPase) is a substrate of OMA1, when OMA1 is activated, it converts OPA1 into a short form of OPA1. CCCP, an inhibitor of mitochondrial oxidative phosphorylation, was used as a positive control. Knockdown of DEL1 blocks CCCP-induced upregulation of ATF4 and CHOP, but does not reduce PG3 or ONC201-induced upregulation of ATF4 and CHOP (Supplementary Figure 1A, 1B). As shown in Supplementary Figure 1A, 1B, the first OMA1 siRNA failed to knock down OMA1. Then we used a second OMA1 siRNA to do knockdown experiments, as shown in Supplementary Figure 1C, 1D, knockdown of OMA1 blocks CCCP-induced upregulation of ATF4 and CHOP, but not PG3 or ONC201. Taken together, these data indicate that activation of HRI by PG3 or ONC201 is not through the OMA1/DELE1 pathway.

It was also reported that ROS and NO (Nitric oxide) prevent heme from binding to HRI, leading to HRI activation [52–54]. Heme oxygenase-1 (HO-1) mediates the first step of heme catabolism, resulting in decreased heme levels in cells [54]. Therefore, we tried to find out whether PG3 activates HRI through ROS, or NO, or HO-1, and ONC201 was used as a positive control since it was previously reported that ONC201 treatment boosts ROS levels [51]. L-NMMA (N-Methyl-L-arginine acetate) is a nitric oxide synthase (NOS) inhibitor and it inhibits all NOS isoforms including nNOS, eNOS, and iNOS. NAC (N-acetylcysteine) is a well-known ROS scavenger. ZnPP (Zinc protoporphyrin) is a potent HO-1 inhibitor. Unfortunately, NAC, or L-NMMA, or ZnPP cotreatments do not attenuate or block PG3- or ONC201-induced upregulation of ATF4 (Supplementary Figure 2A, 2B), which indicates that PG3 or ONC201 do not activate ATF4 through ROS, or NO, or HO-1.

ALAS1 (5′-aminolevulinate synthase 1) catalyzes the first rate-limiting step in heme (iron-protoporphyrin) biosynthesis, which is the condensation of glycine with succinyl-CoA to form δ-aminolevulinic acid [55]. ONC201 treatment leads to potent downregulation and inhibition of ALAS1, indicating that ONC201 inhibits heme biosynthesis (Supplementary Figure 2A, 2B). By contrast, PG3 treatment does not lead to the downregulation of ALAS1 (Supplementary Figure 1A, 1B). UROD (uroporphyrinogen decarboxylase) catalyzes the fifth step in heme biosynthesis [55] and was also included in our western blot experiments. Both ONC201 and PG3 treatments do not have any effects on UROD protein levels (Supplementary Figure 1A, 1B).

**Figure 8:**
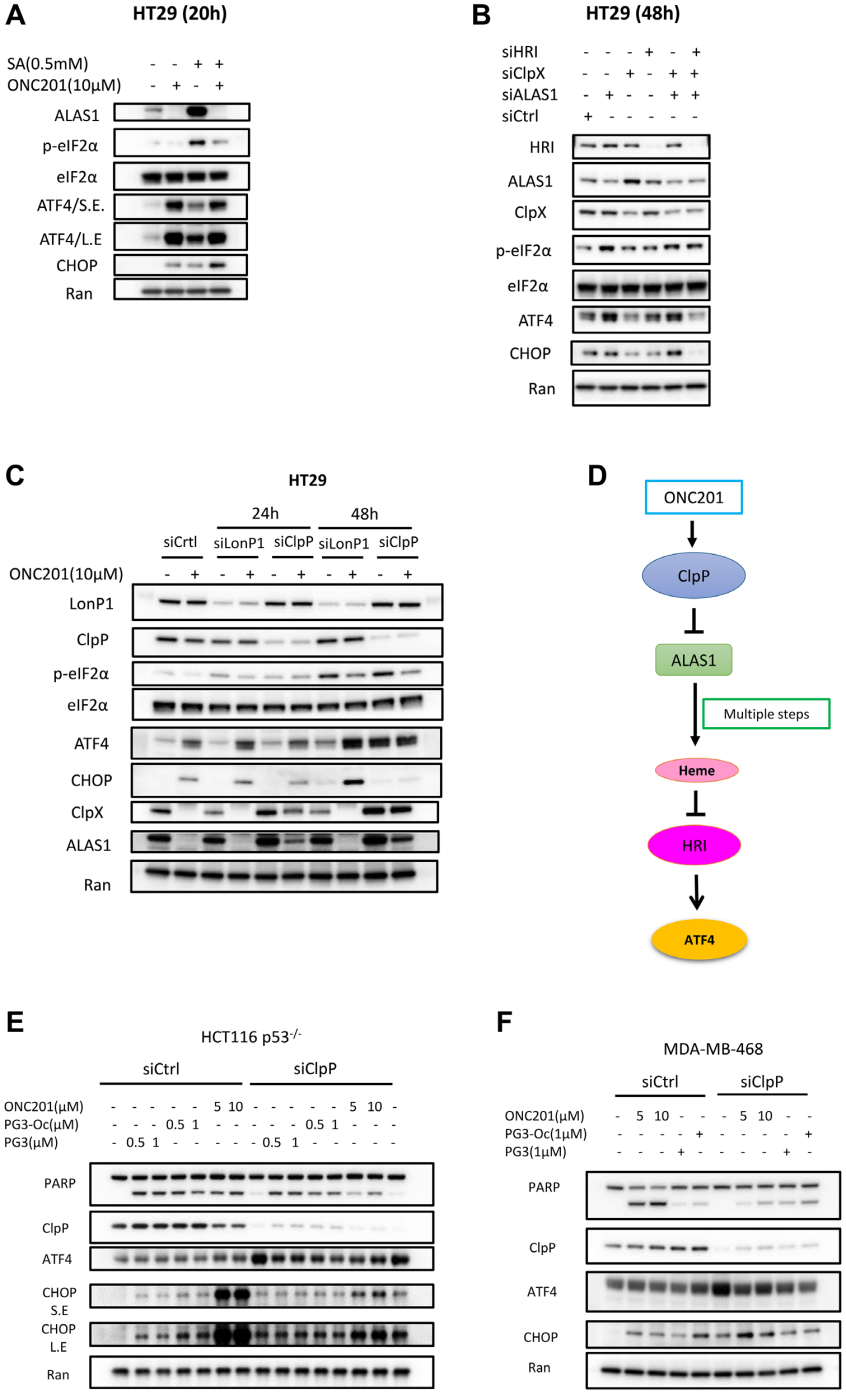
Inhibition of heme biosynthesis leads to upregulation of ATF4. (**A**) HT29 cells were treated with SA and ONC201 for 20 hours. Western blotting was performed using the indicated antibodies. (**B**) HT29 cells were transfected with siCtrl, siHRI, siClpX, siALAS1, siClpX/siALAS1 and siClpX/siALAS1/siHRI for 48 hours. (**C**) HT29 cells were transfected with siCtrl, siLonP1, siClpP for 24 or 48 hours respectively, and then treated with ONC201 for 24 hours. (**D**) Proposed model of ONC201-induced cell apoptosis through ClpP/ALAS1/HRI/ATF4 axis. (**E**, **F**) Silencing of ClpP in HCT116 p53^−/−^ and MDA-MB-468 cells and then treated with PG3, PG3-Oc and ONC201. Western blot was performed using indicated antibodies.

It is well known that downregulation of heme level results in activation of kinase HRI. Hence, we hypothesize that ALAS1 downregulation by ONC201 contributes to HRI activation. Succinylacetone (SA) is a specific inhibitor of δ-aminolevulinic acid dehydratase that catalyzes the second step in heme biosynthesis. It specifically inhibits heme biosynthesis [55]. To test this hypothesis, SA was used as a positive control. HT29 cells were treated with SA and ONC201 respectively. Western blot analysis indicates that SA treatment leads to eIF2α phosphorylation and upregulation of ATF4 and CHOP that is comparable to that of ONC201 (Figure 8A). SA treatment also leads to upregulation of ALAS1 (Figure 8A), which is consistent with previous publications that SA treatment induces upregulation of ALAS1, which is why SA is also known as ALAS1-inducer. ONC201 and SA combination treatment leads to eIF2α phosphorylation and upregulation of ATF4 and CHOP. Surprisingly, ONC201 was able to eliminate SA-induced accumulation of ALAS1 (Figure 8A).

To further confirm these observations, siRNA knockdown was performed. Kardon et al. reported that ClpX (caseinolytic mitochondrial matrix peptidase chaperone subunit X) activates ALAS1 in heme biosynthesis by catalyzing cofactor binding [56]. As shown in Figure 8B, knockdown of ALAS1 induces eIF2α phosphorylation and ATF4 upregulation, however, knockdown of ClpX does not induce upregulation of ATF4 in HT29 cells. Double knockdown of ALAS1 and ClpX leads to upregulation of ATF4 comparable to knockdown of ALAS1 alone, and knockdown of HRI blocks ATF4 upregulation induced by knockdown of ALAS1. Taken together, these data support that ONC201 activates HRI through inhibition of ALAS1.

Mitochondrial ClpP (caseinolytic peptidase P) is a serine protease located in the mitochondrial matrix and LonP1 (mitochondria Lon protease) is another major protease in the mitochondrial matrix. These proteases participate in mitochondrial protein quality control by degrading misfolded or damaged proteins, thus maintaining normal metabolic function. It has been established that ONC201 binds to ClpP and leads to ClpP hyperactivation [51]. It is known that ClpX is a substrate of ClpP and ONC201 treatment leads to its downregulation [57]. ALAS1 is both a substrate of ClpP and LonP1 [56, 58, 59]. To find out whether ClpP activation by ONC201 results in ALAS1 degradation, siRNA knockdown experiments were performed. As shown in Figure 8C, knockdown of ClpP partially rescues ClpX and ALAS1 at the 24-hour timepoint, and almost completely rescues both proteins at the 48 hour timepoint as compared to control siRNA treatment. But knockdown of LonP1 does not rescue ClpX and ALSA1 downregulation compared to control siRNA. These data suggest that ClpP mediates ONC201-induced downregulation of ALAS1.

In short, for the first time, our work demonstrates a novel link between ClpP activation induced by ONC201 treatment and ATF4 upregulation, via the ClpP-ALAS1-HRI pathway (Figure 8D). We further sought to determine the target of PG3 and what signaling pathway leads to PG3-induced activation of HRI.

Knockdown of ClpP attenuates ONC201-induced cell death but not PG3-induced cell death To compare whether siRNA silencing of ClpP will rescues cell death induced by PG3, PG3-Oc or ONC201, ONC201-sensitive HCT116 p53^–/–^ and MDA-MB-468 triple-negative breast cancer cell lines were treated with the three compounds respectively, and then apoptosis marker cleaved-PARP was probed by western blot. As shown in Figure 8E, 8F, knockdown of ClpP did not block the induction of cleaved-PARP by PG3 and PG3-Oc in both cell lines. Silencing of ClpP significantly reduced PARP cleavage-induced by ONC201 in both HCT116p53^–/–^ and MDA-MB-468 cells (Figure 8E, 8F).

Knockdown of ClpP alone results in upregulation of ATF4 (Figure 8E, 8F). This makes sense because Knockdown of ClpP leads to the accumulation of unwanted proteins in the mitochondria, which triggers mitochondrial stress and activation of ATF4. Also, silencing of ClpP potently blocks ONC201-induced upregulation of CHOP in HCT116 p53^–/–^ cells at both 5 and 10 μM of ONC201, but not in MDA-MB-468 cells (Figure 8E, 8F). Taken together, these data support that ClpP is not involved in PG3-induced HRI activation but is responsible for ONC201-induced HRI activation.

## DISCUSSION

A traditional approach to restoring the p53 pathway involves altering mutant p53 protein to the wild-type conformation of p53 through small molecules by either covalent (APR-246) or non-covalent (ZMC1) binding to mutant p53, which restores sequence-specific DNA-binding and transcriptional activation by several p53 point-mutants of either the ‘conformation’ or ‘contact’ type. This approach is p53-dependent. The limits of this approach include: (1) there are thousands of p53 mutations which are not practical to target one by one, (2) this approach cannot target p53 deletion, p53-null, and frame-shift mutations, (3) it has not been previously published that p53 with double mutations, such as human colorectal cancer cell line SW480 (R273H/P309S), could be targeted to restore its wild-type conformation, (4) it is unknown whether some of the compounds directly bind to mutant p53 and mechanisms of restoration of p53 pathway are still undetermined, such as CP31398 and ellipticine, and (5) APR-246, ZMC1, CP31398 and ellipticine also induce cell death in a p53-independent way, which make it complicated to evaluate their anti-cancer effects *in vivo*. To address these issues, we have pursued restoration of p53 pathway signaling in a p53-independent manner. The difficulty of this approach is to find other transcription factors that regulate a subset of p53 target genes, which must include key p53 target genes that regulate cell apoptosis. Such genes are downstream mediators of anti-cancer effects and so another challenge becomes to identify the direct molecular targets of small molecules that by definition are different from p53 itself but activate downstream responses that substitute for p53-deficiency.

Our published paper provides proof-of-concept regarding the feasibility and versatility that a novel compound PG3-Oc restores the p53 pathway in cancer cells in a p53-independent way through transcription factor ATF4 [6]. Our approach with compounds such as PG3-Oc does not predict alteration in mutant p53 protein or p53-specific genomic DNA-binding through mutant p53. Instead, another activated factor ATF4 “restores” p53 target gene activation after drug treatment, essentially bypassing the defective mutated-p53 pathway.

To perform a comparative activation of p53 target genes with PG3 and PG3-Oc, five cancer cell lines with conformation p53-R175H mutation (TOV-112D), contact p53-R273H mutation (HT29), and an isogenic HCT116 cell line with wild-type p53, p53-null and p53-R175H were selected. Four well-known compounds that restore wild-type p53 conformation of mutant p53s and p53-specific DNA-binding, which are APR-246, ZMC1, CP31398, and ellipticine, were selected. Nutlin-3a is used as a negative control. Based on cell viability assays, p53-dependent inhibition after treatment with mutant p53-targeting compounds respectively was observed only at an exact concentration of the specific compound. Above this predetermined concentration, the compounds induce cell death in a p53-independent manner. PG3 and PG3-Oc showed almost the exact same potency against the five cell lines, indicating the chemical modification by removing the ester group and shortening the long hydrophobic sidechain of PG3-Oc did not affect its anticancer activity. Importantly, we noticed that this modification reduced PG3-Oc toxicity to normal cells, therefore, PG3 also has an increased therapeutic index as compared to PG3-Oc.

p53-responsive reporter assays using the SW480 (R273H/P309S) cell line show PG3 does not activate the reporter activity but significantly induces upregulation of DR5, PUMA, and p21 in the five cell lines, including the HCT116 p53^–/–^ cell line. siRNA knockdown of ATF4 blocks PG3-induced upregulation of PUMA, NAG-1/GDF15, and p21. siRNA knockdown of PUMA prevents PG3-induced apoptosis. Taken together, these data indicate that PG3 treatment induces upregulation of p53 target genes through ATF4 in a p53-independent way and induces PUMA-mediated apoptosis. CP-31398 and ellipticine can activate the p53-responsive reporter activity significantly, suggesting the two compounds might restore wild-type conformation and p53-specific DNA-binding of p53 with the double mutations or upregulate p53 family members p73 or p63. ZMC1 and APR-246 do not activate the reporter activity, suggesting that the two compounds are not able to restore wild-type conformation of the mutant p53 (R273H/P309S) or activate p73 or p63.

PG3 and PG3-Oc upregulate p21, PUMA, and DR5 consistently in five tested cancer cell lines with varying p53 status through ATF4 in a p53-independent way. However, the mutant p53-targeting compounds induce expression of the three p53 target genes in a highly variable and cell type-dependent way. For example, CP31398-induced upregulation of DR5 is cell-type dependent, whereas APR-246, ZMC1, and ellipticine do not induce upregulation of DR5 in the five cell lines. Interestingly, ZMC1 activates ATF4 in the five cell lines to the same extent as PG3 and PG3-Oc, indicating that ZMC1 treatment also triggers the ISR, which may contribute to its anti-cancer activity. CP-31398 induces upregulation of ATF4 in a cell type-dependent manner. However, APR-246 and ellipticine do not induce upregulation of ATF4 in the five tested cell lines, indicating that these two compounds do not trigger the ISR at the concentrations used in our experiments.

Dissection of the molecular mechanisms led us to identify ATF4 as a key regulator, which mediates the expression of p53 target genes in p53 mutant and null cancer cell lines after PG3 treatment. We propose a model (Figure 7G) in which activation of ATF4 through the IRS via kinase HRI by PG3 leads to upregulation of PUMA, p21, and NAG-1/GDF15. We discovered that PUMA mediates activation of caspase-8 in HT29 cells, but the mechanism has not been identified so far. Interestingly, PUMA also mediates activation of both caspase-8 and caspase-9 in SW480 cells (Figure 6C). It is possible that the activation of caspase-10 mediates apoptosis in SW480 cells because a caspase-10 specific inhibitor potently blocks the cleavage of caspase-3 and PARP. ATF4 shares a subset of p53 target genes involved in cell cycle arrest, autophagy, and apoptosis [6]. Recently, using a multiomics approach, it was discovered that ATF4 is a key regulator of and a biomarker of mitochondria stress response in humans [60]. Mitochondrial protease ClpP (caseinolytic protease P) activation by ONC201 results in the ISR and an increase in ATF4 [51]. However, the mechanisms connecting mitochondrial stress to the ISR were unknown until recently when it was identified for the first time that mitochondrial stress leads to the ISR and ATF4 activation by an OMA1-DELE1-HRI pathway [25, 26]. These observations suggest that ATF4 is a critical node for responding to various intrinsic and extrinsic stresses and that ATF4 regulates cell fate. In the future, more research is needed to understand how ATF4 regulates p53 target genes, and how many p53 target genes have ATF4 binding sites, etc. Also, there may be other transcription factors that regulate a particular subset of p53 target genes.

In summary, the insights from our work could provide the basis for novel drug discovery and development of compounds that treat p53-mutated and p53-null cancers through the induction of ATF4 in a p53-independent manner.

## MATERIALS AND METHODS

### Cell lines and reagents

P53-mutant cell lines: HT29 (R273H), TOV-112D (R175H), SW480 (R273H/P309S), DLD-1 (S241F), P53 wild-type cell lines: HCT116; P53-null cell line: HCT116 p53^–/–^. HFF-1, MRC5, and MEF cells were purchased from ATCC. HT29, SW480, DLD-1, and HCT116l cell lines were purchased from Fox Chase Cancer Center cell culture facility. HCT116 p53^–/–^ cell lines were from the Vogelstein laboratory at Johns Hopkins. HT29-PUMA^–/–^ cell line was created in our laboratory [6]. PERK^−/−^, and GCN2^−/−^ MEFs cells were shared by S. Kimball and N. Dolloff, respectively. Cells were routinely checked for mycoplasma and all cell lines underwent STR authentication. Chemicals: Prodigiosin was from NCI (National Cancer Institute); ZMC1 (MedChemExpress); APR-246, Nutlin-3a, GSK2606414 and obatoclax (Selleckchem); Caspase 10 inhibitor (R&D Systems); CP-31398 and thapsigargin (Tocris Bioscience); Ellipticine (Cayman Chemicals), and ONC201, ONC206 and ONC212 (Chimerix, Durham, NC, USA).

### Cell viability assay

Cells were seeded in 96-well plates (6 × 10^3^ cells/well). Cells were treated with different concentrations of compounds or dimethyl sulfoxide (DMSO) as a control for 72 hours. The cell viability was assessed by CellTiterGlo bioluminescent cell viability assay (Promega), following the manufacturer’s protocol. Bioluminescence imaging was measured using the IVIS imager. The percentage of cell viability (mean ± SEM) at each dose was calculated against the respective DMSO control. The IC_50_ values were determined from the sigmoidal dose-response curves using GraphPad Prism.

### p53-responsive reporter assay

p53-mutant SW480 human colon cancer cells, stably expressing a p53-responsive luciferase reporter [61], were used to assay functional restoration p53 pathway-dependent transcription in mutant p53-expressing tumor cells. Cells were seeded in 96-well plates (5 × 10^4^ cells/well) and were treated with small-molecule compounds for 6 and 15 h, respectively. Then, D-luciferin was added to each well at a final concentration of 100 μg/mL, and cells were imaged by using an IVIS Imaging System (Xenogen) to detect luciferase activity.

### Western blotting

After treatment, protein lysates were collected for Western blot analysis. A total of 15 μg of protein was used for SDS-PAGE. After primary and secondary antibody incubations, the signal was detected by a chemiluminescence detection kit, imaged by Syngene (Imgen Technologies). Antibodies for NAG-1 (GDF15) and P53 were from Santa Cruz Biotechnology; for caspase 8, cleaved caspase 8, caspase 9, caspase 3, cleavage PARP, eIF2α, p-eIF2α (Ser51), CHOP, ATF4, DR5, and PUMA were from Cell Signaling Technology. Noxa and p21 were from Calbiochem. Ran was from BD Biosciences.

### siRNA knockdown

Knockdown experiments were performed by transfecting either 80 pmoles of indicated siRNA(s), or scramble siRNA using RNAiMAX (Invitrogen), following the manufacturer’s protocol. Transfected cells were treated with PG3, 24 hours post-transfection. The control scrambled siRNA and siRNAs for human caspase 8, Caspase 9, NAG-1/GDF15, PKR, HRI, ATF4, and PUMA were purchased from Santa Cruz Biotechnology.

### Flow cytometry

Cell Death Analysis—Zombie Violet staining and flow cytometry were used to determine the degree of cellular death. Cells were seeded at 5 × 10^5^ cells/well in 6-well plates. Cells were treated with PG3 for 48 and 72 hours. Cells were harvested, and stained by Zombie Violet dye, then flow cytometry was performed as manufacture’s protocol (CytoFlex, Beckman Coulter, Brea, California).

## SUPPLEMENTARY MATERIALS


