# Supplementary material for: Integrated stress response (ISR) activation and apoptosis through HRI kinase by PG3 and other p53 pathway-restoring cancer therapeutics
Source: Oncotarget. 2024 Sep 17;15:614–33. doi: 10.18632/oncotarget.28637 (PMC11407758; doi:10.18632/oncotarget.28637)
Supplement: Supplementary file 1 [file oncotarget-15-28637-s001.pdf]

Integrated stress response (ISR) activation and apoptosis through HRI kinase by PG3 and other p53 pathway-restoring cancer therapeutics

SUPPLEMENTARY MATERIALS

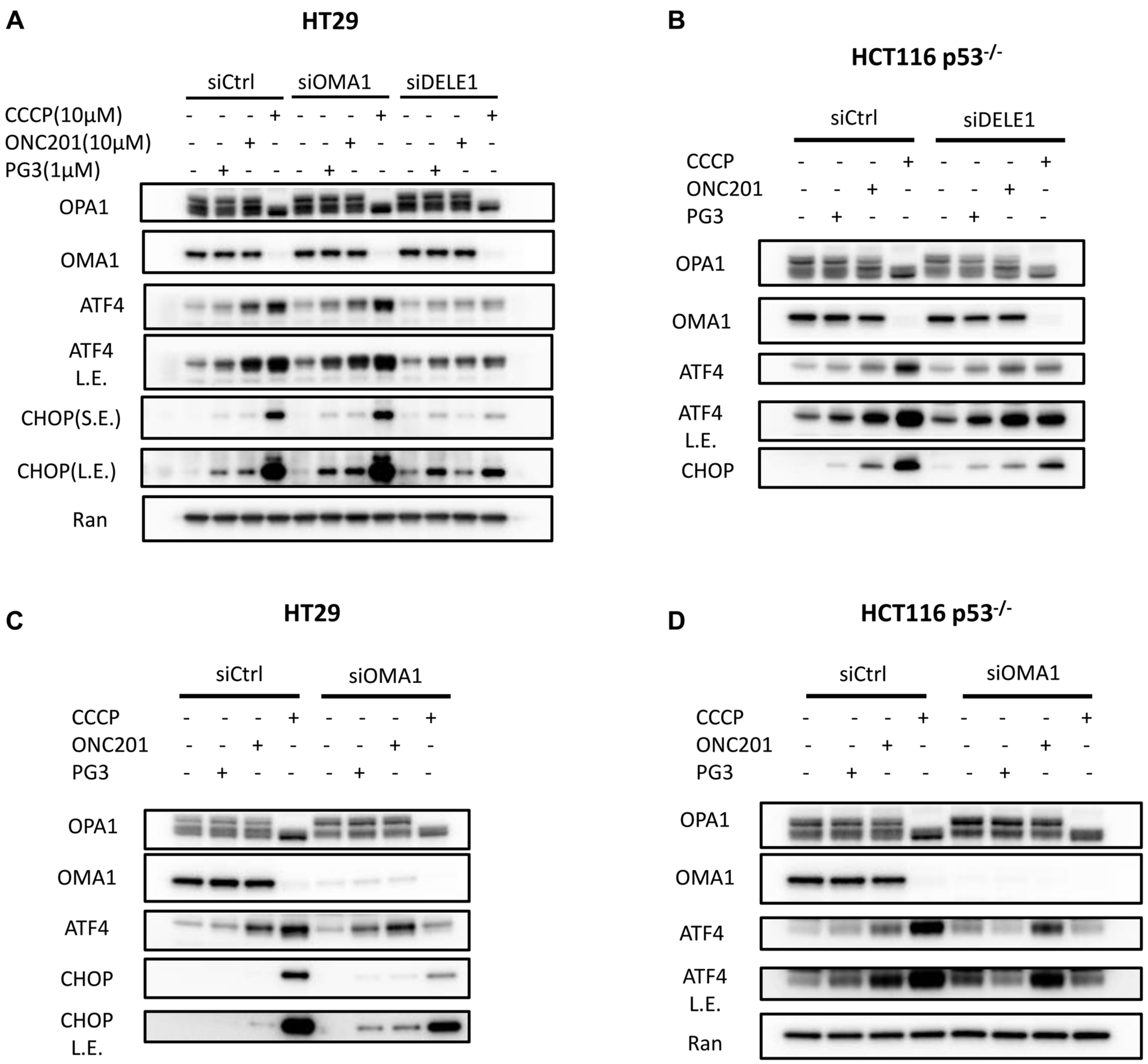

Supplementary Figure 1: OMA1/DELE1/HRI pathway does not mediate HRI activation. (**A**) HT29 cells were transfected with siCtrl, siOMA1 and siDELE1 for 48 hours, and then treated with compounds CCCP, ONC201 and PG3, respectively. (**B**) HCT116 $p53^{-/-}$ cells were transfected with siCtrl, and siDELE1 for 48 hours, and then treated with compounds CCCP, ONC201 and PG3, respectively. (**C**) HT29 cells were transfected with siCtrl, and siOMA1 for 48 hours, and then treated with compounds CCCP, ONC201 and PG3, respectively. (**D**) HCT116 $p53^{-/-}$ cells were transfected with siCtrl, and siOMA1 for 48 hours, and then treated with compounds CCCP, ONC201 and PG3, respectively.

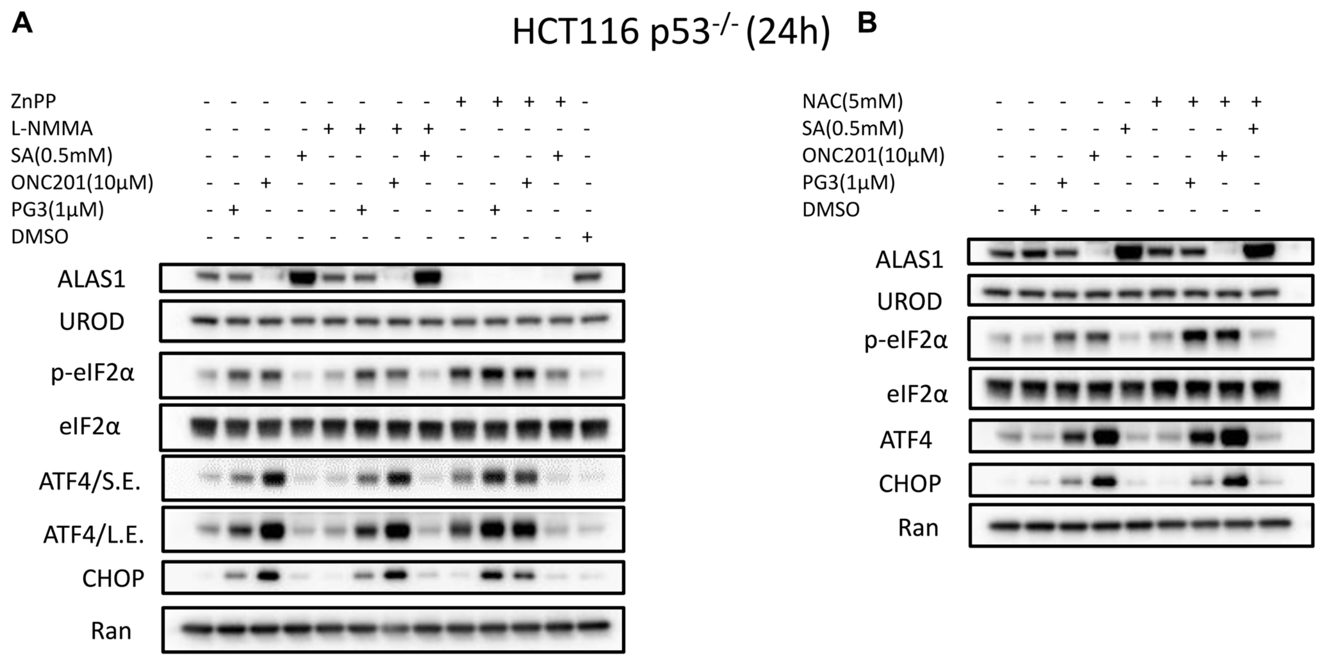

Supplementary Figure 2: ROS and NO are not responsible for PG3-induced HRI activation. (**A**, **B**) cells were pre-treated with ZnPP, L-NMMA, SA and NAC for 2 hours respectively, and then ONC201 or PG3 was added to the medium for 24 hour treatment. Western blot was performed using indicated antibodies.
